# Accuracy of Sonographic Airway Parameters in Difficult Laryngoscopy Prediction: A Prospective Observational Cohort Study from Central India

**DOI:** 10.4274/TJAR.2023.231217

**Published:** 2023-10-24

**Authors:** SK Parameshwar, Sunaina Tejpal Karna, Vaishali Waindeskar, Harish Kumar, Pooja Singh, Saurabh Saigal

**Affiliations:** 1 Department of Anaesthesiology and Critical Care, All India Institute of Medical Sciences, Bhopal, India

**Keywords:** Airway management, airway ultrasonography, difficult airway screening test, difficult laryngoscopy, preoperative airway assessment

## Abstract

**Objective:**

Though airway ultrasonography (USG) is used to assess difficult laryngoscopy (DL), there is still ambiguity about approach followed and parameters assessed. There is need of a simple, stepwise sonographic assessment with clearly defined parameters for DL prediction. The primary objective of this study was to find diagnostic accuracy of sonographic parameters measured by a stepwise Airway-USG in DL prediction (DLP).

**Methods:**

This prospective, observational cohort study was done in 217 elective surgical adult patients administered general anaesthesia with tracheal intubation using conventional laryngoscopy from 1^st^ May 2019 to 31^st^ July 2020, after ethical approval. A sagittal Airway-USG was done using 2-6 Hz transducer in three steps specifying probe placement and head position. Demographic, clinical and Airway-USG measurements were noted. Correlation of the clinical/sonographic parameters was made with Cormack-Lehane score on DL. After receiver operating characteristic curve plotting, the sensitivity, specificity, positive predictive value, negative predictive value (NPV) of DL was calculated for each parameter using open-epi software.

**Results:**

DL was observed in 19/217 patients. Airway-USG parameters of skin to epiglottis distance >2.45 cm, hyomental distance with head extension <5.13 cm, head neutral <4.5 cm, their ratio <1.18, maximum tongue thickness >3.93 cm and maximum skin to tongue distance >5.45 cm were statistically significant in predicting DL. DLP score with presence of >3 positive parameters showed 98% specificity, 98% NPV and 96% diagnostic accuracy to predict DL.

**Conclusion:**

DLP score derived from Airway-USG may be used as a screening and diagnostic tool for DL.

**Main Points**

• Three-step Airway-ultrasonography (USG) can be used to assess occipital-atlantoaxial extension, submandibular space compliance, epiglottis position and tongue size.

• Difficult laryngoscopy (DL) predictor (DLP) score can be derived from measured parameters of Airway-USG.

• DLP score has a good screening and diagnostic potential to predict DL when more than 2 and 3 parameters, respectively are positive.

## Introduction

Airway associated complications are the most common anaesthesia-related adverse outcomes.^1^ Intubation failure is usually attributed to difficult laryngoscopy (DL).^2,3^ The low sensitivity, high inter-observer variation of morphometric screening tests like Mallampati classification, upper lip bite test, thyromental distance, cervical spine movements has led to continued search for more accurate airway examination tool.^4,5^ Airway-ultrasonography (USG) is a non-invasive, portable bedside method which can visualize anatomical airway structures, confirm placement of endotracheal (ET)/double-lumen tube and guide invasive procedures like percutaneous tracheostomy and cricothyroidotomy.^6,7,8,9,10^

A recent meta-analysis has highlighted the heterogeneity in performance of airway sonography.^11,12,13,14^ There needs to be more literature on accuracy of a step-wise sonographic airway assessment to predict DL better. With this research gap, we conducted this study with the primary objective of studying accuracy of sonographic airway assessment using a three-step approach of protocolized stepwise Airway-USG examination in prediction of DL seen by Cormack-Lehane (CL) Scoring system in patients administered general anaesthesia with ET intubation for elective surgery.

## Methods

### Subjects and Methods

This single-centre, prospective observational study was conducted in an academic tertiary care hospital in Central India in American Society of Anesthesiologists Physical Status (ASA-PS) I-III patients, aged 18-70 years, undergoing elective surgery under general anaesthesia with ET intubation from 1^st^ May 2019 to 31^st^ July 2020. Ethical clearance was given by the Institutional Ethics Committee (IHEC-LOP/2019/MD0049). All study participants gave written informed consent. We excluded patients with any airway abnormality preventing the use of clinical screening tests and Airway-USG like head and neck surgery/trauma/tumors/burns/scars/radiotherapy injuries/neck abscess/hematoma/beard, medical conditions like rheumatoid arthritis, ankylosing spondylitis, pregnancy, extreme obesity [body mass index (BMI) ≥40 kg m^2-1^], previous history of DL and where laryngoscopy was not part of anaesthesia plan.

### Data Collection

On the preoperative day, a trained anaesthesiologist collected the demographic variables and clinical airway parameters like inter-incisor gap, modified Mallampati score and thyromental distance. A single trained study investigator, blinded to the clinical airway parameters, performed the Airway-USG examination using a 2-6 Hz curvilinear transducer of the SonoSite M Turbo portable ultrasound machine. During Airway-USG, all patients were positioned supine with mouth closed and were instructed to keep their tongue relaxed and touch the lower incisors, without phonation or deglutination.

**Figure 1:**
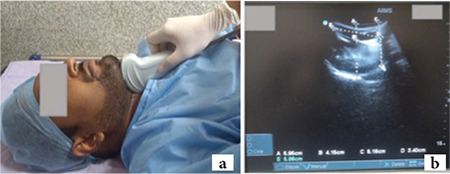
a) First step Airway-USG-patient head in neutral position with curvilinear transducer placed in suprahyoid region at the midline in sagittal plane, adjusted to bring the hyoid bone, muscles of floor of the mouth, the entire tongue and mentum in one frame. b) Ultrasonographic image at first step. A=Skin to tongue distance-maximum, B=Tongue thickness-maximum, C=Skin to tongue distance at base, D=Tongue thickness at base of tongue, E=Hyomental distance in neutral (Note hyperechoic air-mucosa interface at dorsum of tongue). USG, ultrasonography.

Several upper airway anatomical components influence the glottic view during laryngoscopy. Tongue and oral cavity volume, submandibular space compliance, epiglottis and extension at occipito-atlanto-axial joint are important. To assess and quantify these components ultrasonographically, yet keep it simple to perform, we proposed a three-step approach of protocolized step-wise Airway-USG examination. The three steps of as follows:

**Step 1:** With the patient’s head in a neutral position, the transducer was placed in the midline of suprahyoid region in sagittal plane, as shown in Figure 1a, and adjusted to bring the hyoid bone, muscles of the floor of the mouth (geniohyoid and mylohyoid), the entire tongue and mentum in a single frame (Figure 1b). The following parameters were measured.

Tongue thickness was measured at the base of tongue (TT_B_) and at a maximum vertical distance (TT_M_), from the tongue’s dorsum to the geniohyoid muscle’s dorsum.

Skin-to-tongue distance was measured at the base of tongue (STD_B_), and a maximum vertical distance (STD_M_), from the dorsum of the tongue to the skin surface.

The hyomental distance also measured in a neutral position (HMD_N_) from the hyoid bone’s upper border to the mentum’s lower border.

**Figure 2:**
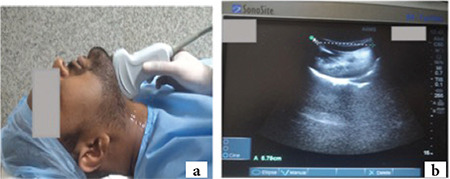
a) Second step Airway-USG-patient head in extended position with curvilinear transducer placed in suprahyoid region at midline in sagittal plane, adjusted to bring the hyoid bone and mentum in one frame. b) Ultrasonographic image at second step. A=Hyomental distance in extension. USG, ultrasonography.

**Step 2:** The patient’s head was extended (Figure 2a) without changing the probe position. Hyomental distance in extension (HMD_E_) was measured from hyoid bone’s upper border to mentum’s lower border (Figure 2b).

**Figure 3:**
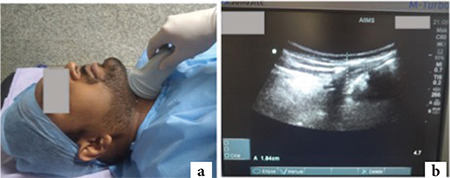
a) Third step Airway-USG-patient head in neutral position with curvilinear transducer placed in Infrahyoid region at the midline in sagittal plane (Note the transducer is moved caudally from step-1 to trace the epiglottis still keeping the hyoid bone in plane). b) Ultrasonographic image at third step. A=Distance from skin to posterior surface of epiglottis, measured just below hyoid bone (Note Hyperechoic air-mucosa interface at posterior surface of epiglottis). USG, ultrasonography.

**Step 3:** With the head back in a neutral position, the transducer was slowly moved caudally in the midline to the infrahyoid region, keeping the hyoid bone in frame (Figure 3a), to trace the entire length of epiglottis, which appeared as a hypoechoic structure with hyperechoic air-mucosa interface on its posterior surface. Distance from skin to epiglottis (DSE) was measured just below the hyoid bone from skin surface to the posterior surface of epiglottis (Figure 3b).

On the day of surgery, standard institutional protocols were followed for induction of general anaesthesia with ET intubation done by an independent conventionally trained anaesthesiologist with more than 5 years of experience using Macintosh laryngoscopes of appropriate size blinded to preoperative airway sonography findings. The CL grading was noted.^15^

For each case, the study’s end point was the difficulty in laryngoscopy judged by the CL grading, where Grades 1 or 2 and Grades 3 or 4 were considered easy and DL, respectively. The demographic, clinical and Airway-USG parameters were compared between easy and DL patients.

### Statistical Analysis

Based on previous studies, the sensitivity of USG parameters was reported from 65% to 85% (average 75%) and clinical screening tests was reported from 20-62% (average 41%).^16,17^ To estimate at least 30% higher sensitivity of USG over clinical parameters with 80% power to detect this change, considering a prevalence of 9.5% to 12% (average of 11) DL for the Indian population, with 95% confidence interval (CI), the estimated sample size calculated using PASS software was 200. We assumed an attrition rate of 10% and calculated the final sample size as 220 patients.

Data was entered, cleaned, and coded in Microsoft Excel 2013. Data was analysed using IBM Statistical Package for the Social Sciences version 23. The Shapiro-Wilk method was used to test the distribution normalcy of numerical variables and presented as mean [standard deviation (SD)] when normally distributed, while non-normally distributed variables presented as median [interquartile range (IQR)]. Categorical variables were expressed as absolute numbers and percentages. Ratios were expressed as values and their 95% CI.

Pearson’s chi-square and Fisher’s exact tests were used as significance tests for the association between categorical variables. Using Levene’s test for equality of variances, numerical variables were checked for homogeneity between the difficult and easy laryngoscopy groups. Independent samples t-test and ANOVA test were used as tests of significance for homogenous numerical variables, while Mann-Whitney U test was used as test of significance for non-homogenous numerical variables. Correlation analysis was performed using the Pearson test. Receiver operating characteristic (ROC) curves were plotted and optimal cut-off values were determined using Youden’s index.

Four derived parameters were calculated from the measured values.

**Hyomental distance ratio (HMD_R_)** is defined as the ratio of HMD_E_ divided by HMD_N_ head position.

**Delta_HMD** is defined as percentage change in Hyomental distance during Occipito-Atlanto-Axial joint (Neck) extension.

$\mathrm{\Delta HMD}=[\frac{\mathrm{HMDE}–\mathrm{HMDN}}{\mathrm{HMDE}}]X100$

**R1** defined as ratio of tongue thickness (TT_M_) to skin to tongue distance (STD_M_) at maximum tongue width.

**R2** defined as ratio of tongue thickness (TT_B_) to skin to tongue distance (STD_B_) at base of the tongue.

### “Difficult Laryngoscopy Prediction (DLP)” Scoring System

Since DL is influenced by complex upper airway anatomy, a composite DLP score was developed using statistically significant USG parameters measuring different static and dynamic upper airway components. Diagnostic parameters such as sensitivity, specificity, positive predictive value (PPV), negative predictive value (NPV), likelihood ratio (LR) and diagnostic accuracy were calculated for individual and composite parameters using open-epi software.

## Results

**Figure 4:**
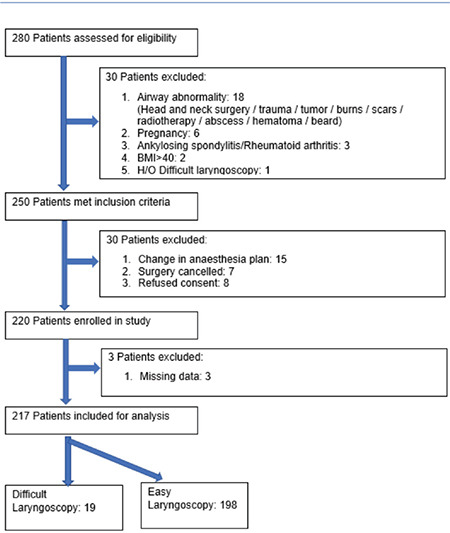
Study flow chart and outcome. BMI, body mass index.

During the study period, 280 patients were assessed for eligibility, 220 patients were enrolled, and data analysis was possible in 217 patients. (Figure 4). The median age of this study population was 37 (IQR: 22) years, 60% of them female. The study included general surgical (25.8%), gynecological (17.1%), neuro-surgical (13.8%) and onco-surgical (10.6%) patients operated under general anaesthesia. We observed an 8.8% incidence of DL (19/217). The measured and derived sonographic parameters were noted to have a normal distribution, and homogenous variance except TT_B_, STD_B_ and R2.

### Association of DL with Demographic and Clinical Airway Parameters

Patients with DL were observed to have higher age [43 (IQR: 16) years vs 36 (IQR: 20) years, P=0.002] and BMI [26.62±3.13 (95% CI: 25.11-27.12) kg m^2-1^ vs 22.77±3.91 (95% CI: 22.31-23.31) kg m^2-1^, P=0.002] in comparison to those with easy laryngoscopy.

MMP and TMD were the only clinical test observed to be statistically significant in patients with DL. Though both parameters showed poor sensitivity, the specificity was good (Table 1).

### Association of DL with Protocolized Stepwise Airway-USG Examination Parameters

Amongst the measured parameters, HMD_N_, HMD_E_, skin-to-tongue distance maximum (STD_M_), tongue thickness maximum (TT_M_), and DSE were statistically significant in differentiating easy and DL. Pearson correlation analysis showed a strong positive correlation between DL and DSE (r=0.71, *P* < 0.001), moderate negative correlation between DL and HMD_E_ (r=-0.42, *P* < 0.001), small correlation between DL and STD_M_ (r=0.27 *P*=0.01) but minimal correlation between DL and TT_M_, HMD_N_. Amongst the derived, HMD_R_ and delta hyomental distance (Delta_HMD) were statistically significant in differentiating easy and DL. Mean±SD, area under the ROC curve, optimal cut-off value along with their sensitivity, specificity and odds ratio of statistically significant measured and derived variable is mentioned in Table 2 and Table 3, respectively.

One-way ANOVA test and post-hoc analysis (using Dunnett’s t3 multiple comparisons of means) revealed two Airway-USG parameters, namely HMD_E_ and DSE, exhibited statistically significant difference between different CL grades (Table 4).

### Predictor of Difficult Laryngoscopy on Logistic Regression

Multivariate logistic regression showed DSE, HMD_E_, STD_M_ and Delta_HMD were independent predictors of DL, their cut-off values were used to develop the Difficult Laryngoscopy Prediction Score. Each of them scored 1 and 0 for satisfying and not satisfying the cut-off criteria, respectively. DLP score=DSE + HMD_E_ + STD_M_ + Delta_HMD. The diagnostic profile of DLP score ≥2 and ≥3 shown in Table 5.

## Discussion

Several anatomical and pathophysiological components, independently or in combination, can influence the laryngoscopic view. The main anatomical structures obscuring the glottic vision are the tongue, hyoid bone, and epiglottis.^18^ Extension at Occipito-Atlanto-Axial (OAA) joint during laryngoscopy brings the oral axis in near alignment with laryngopharyngeal axes, aiding the glottic vison.^19^ The morphometric screening tests investigate one or a few of these components, hence need better sensitivity. A meta-analysis by Shiga et al.^5^ have confirmed their poor sensitivity with fair specificity. Our results for modified Mallampati score, thyromental distance, and inter-incisor distance were consistent with Shiga et al.^5^ study.

USG has been studied to visualize and quantify upper airway anatomical structures with good precision.^11,12,17^ In our study, we have demonstrated the accuracy of a simple, three step approach of protocolized step-wise Airway-USG examination in anticipating DL. The measured sonographic parameters of HMD_N_, HMD_E_, skin-to-tongue distance at a maximum vertical distance from the dorsum of the tongue (STD_M_), tongue thickness at maximum vertical distance from the dorsum of tongue (TT_M_), DSE and the derived values of HMD_R_, Delta_HMD were significantly associated with DL.

### Hyomental Distance-related Parameters

Hyomental distance in extension (HMD_E_) is an indirect estimate of submandibular space compliance.^1,20^ Large submandibular compliance allows easy compression of the tongue’s bulk, facilitating glottic vision during laryngoscopy. The USG measured HMD_E_ (5.10±0.53 cm for DL) was statistically significant in differentiating easy and DL groups in our study, and the results were consistent with Wojtczak^21^ (<5.20±0.58 cm for DL) results. Lower HMD_E_ in Petrisor et al.^22^ (<4.9±0.22 cm for DL) can be implicated in the high BMI (>40 kg m^2-1^) of their study population.

During head extension at the OAA joint, the mandible moves away from the hyoid bone, whereas the stylohyoid ligament limits the movement at hyoid bone. Thus, the HMD_R_ was proportional to OAA extension.^23^ Sonographic HMD_R_ cut-off observed in our study (<1.18, 73% sensitivity, 65% specificity for DL) was comparable with HMD_R_ values assessed ultrasonographically by Petrisor et al.^22^ (<1.24, 86% sensitivity and 72% specificity) and clinically by Huh et al.^24^ (<1.2, 88% sensitivity and 60% specificity). Since the measured distances are displayed in millimeters, sonographic HMD_R_ values confer good precision over clinical parameters, even in obese patients.^22,25^

Delta_HMD, defined as a percentage change in hyomental distance during OAA extension, is mathematically a better indicator than HMD_R_ for OAA extension. Delta_HMD < 18% indicates that at the end of complete OAA extension, the proportional change in hyomental distance is less than 18%, showing inadequate OAA extension, and DL may be anticipated.

### Tongue Related Parameters

Anatomically, tongue is the largest structure in the oral cavity, obscuring the line of sight during laryngoscopy. Quantifying the tongue size or its volume for the oral cavity can predict DL, as shown by Mallampati et al.^26^. Measuring the tongue and oral cavity volume using 2-dimentional USG was tried by Wojtczak et al.^21^ and Andruszkiewicz et al.^27^ using complex measurements and calculations, but failed to prove their significance in anticipating DL.

To circumvent these complex measurements and calculations, we hypothesized tongue thickness in the sagittal plane at its maximum thickness (TT_M_) and its base (TT_B_) as an indirect indicator of tongue volume. We also measured the distance from skin to dorsal surface of tongue at same points as skin-to-tongue distance maximum (STD_M_) and skin-to-tongue distance at base (STD_B_), respectively, representing the oral cavity volume. Their ratios, R1 (STDM/TTM) and R2 (STDB/TTB), were derived to quantify the tongue volume for oral cavity volume at maximum tongue thickness (R1) and at tongue base (R2). Despite good correlation of these tongue-related parameters with the MMP score, only TT_M_ and STD_M_ could anticipate the DL. However, their ratio R1 failed to express its significance. This failure can be attributed to the two-dimensional representation of tongue volume for the oral cavity.

Even though tongue and floor of the mouth are anatomically two distinct components of the oral cavity, USG measured tongue thickness at its maximum dimensions by Yao and Wang^28^ (>6.2 cm±0.5 for DL) and Yadav et al.^29^ (>6.1 cm IQR: 1.04 for DL) also included the floor of mouth thickness (equivalent to STD_M_ of our study). These results were comparable with STD_M_ of our study results (>5.75±0.32 cm for DL). The imperceptible difference in the results can be attributed to the of head positioning while performing sonography (extension position in their study vs. neutral position in our research). To extend the application of these tongue-related parameters in emergency and intensive care unit patients where the freedom for head extension is often limited, we preferred a head-neutral position over head-extended position.

### Epiglottis Related Parameters

Laryngoscopy aims at lifting the epiglottis. With the increase in soft tissue in the anterior neck, the angle made by the epiglottis with the thyroid cartilage increases, making glottic visualization more difficult, corroborating with the DSE.

USG measurement of anterior neck soft tissue can be performed at the level of hyoid bone, epiglottis, vocal cords and suprasternal notch.^29,30,31,32,33^ When measured at an epiglottic level as DSE, the advantage of indirect quantification of thyroid-epiglottic angle is added. As DSE gradually increases from thyroid to the hyoid bone, we preferred the hyoid bone as an anatomical landmark and measured DSE just below the hyoid bone, to maintain uniformity among measured values. DSE measured just below hyoid bone showed the highest individual sensitivity and specificity amongst all USG-measured parameters in our study.

Our results of DSE (>2.60±0.31 cm for DL) are comparable with Ni et al.^30^ (>2.59±0.41 cm for DL) and Wu et al.^31^ (>2.39±0.34 cm for DL), the indiscernible difference in the results can be due to East Asian ethnicity of their study population. Yadav et al.^29^ (>1.84±0.39 cm for DL) measured DSE at the midpoint of the thyrohyoid membrane. They excluded the epiglottis in DSE measurement, thus explaining the lower DSE value in contrast to our study. Pinto et al.^32^ depicted higher DSE value (>2.82±0.44 cm for DL), as they averaged the measured DSE values at the central axis, the right and left extremity of the epiglottis. The differences in the results can also be attributed to the European ethnicity and higher BMI of their study population.

### Difficult Laryngoscopy Prediction Score

Since DL is influenced by complex airway anatomy involving both static and dynamic components of the upper airway, the diagnostic accuracy of a test could be improved by investigating multiple factors affecting DL. The composite DLP score combines 4 crucial anatomical aspects of DL-DSE for anterior neck soft tissue thickness and thyroid-epiglottic angle, HMD_E_ for submandibular compliance, STD_M_ for tongue and floor of mouth thickness and Delta_HMD for OAA joint extension.

With a 100% sensitivity, 100% NPV, LR- 0.01 and 81% DA, the DLP score ≥2 can be employed as a screening test for DL, thus warning the intubating team about the possibility of DL. DLP score ≥3 had a 98% specificity, 79% PPV, LR+ 39 and 96% DA for DLP and can be employed as a diagnostic test in anticipating DL.

### Strengths and Limitations

The main strength of our study is the simplified three-step Airway-USG assessment method, which may be used in future studies to decrease heterogeneity in the sonographic airway parameters assessed. It systematically examines both static and dynamic components of airway anatomy responsible for DL with good precision. Second, we have highlighted the diagnostic accuracy of the composite DLP score derived for the first time in our study, which encompasses four independent anatomical factors responsible for DL.

Our study has many limitations.

- It is a single center study with limited patients.

- Due to the low incidence of DL, the two study groups had an unequal sample size, which may have impacted the diagnostic profile of the USG parameters.

- We excluded patients with known anticipated DL, like pregnant, morbidly obese, and patients with airway anatomical abnormalities to avoid confounding factors.

- In our study, we never encountered someone with MMP4 score (large tongue to oral cavity ratio); this might have underscored the tongue related USG parameters in anticipating DL.

- We did not have a USG parameter to measure mouth opening, hence lacking complete independence of protocolized step-wise Airway-USG examination in anticipating DL.

## Conclusion

Direct laryngoscopy predictor score derived from a three-step sonographic airway assessment may be utilized as a screening and diagnostic tool for DLP in patients undergoing elective surgery to avoid unanticipated difficult airway. We recommend further studies in different populations to validate the DLP score derived in our study.

## Figures and Tables

**Table 1:**
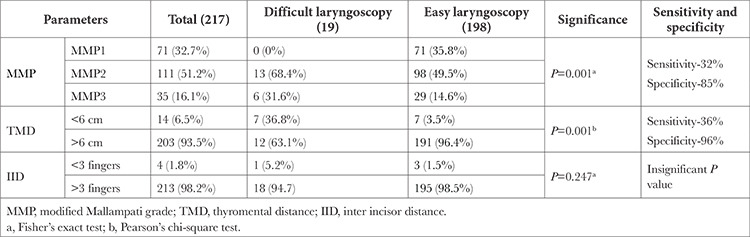
Comparison of Morphometric Difficult Airway Screening Tests in Difficult and Easy Laryngoscopy Groups

**Table 2:**
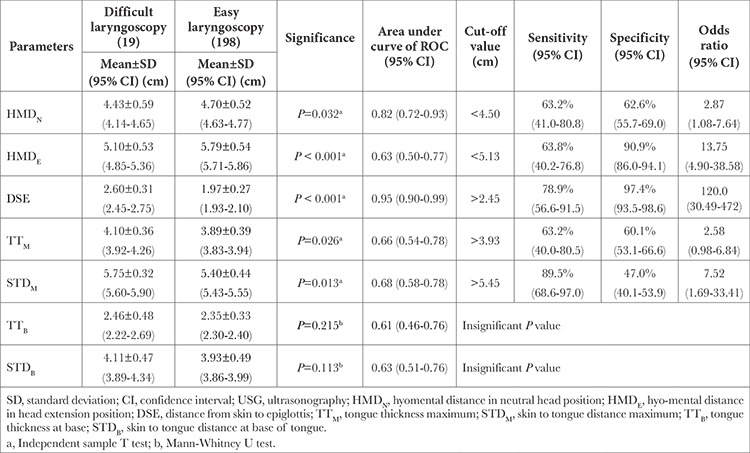
Comparison of Measured USG-Airway Parameters in Difficult and Easy Laryngoscopy Groups

**Table 3:**
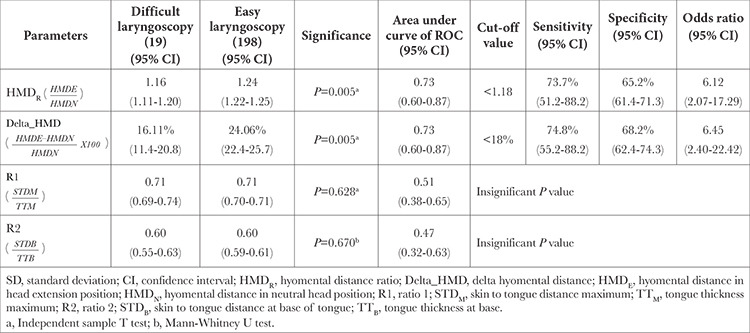
Comparison of Derived USG-Airway Parameters in Difficult and Easy Laryngoscopy Groups

**Table 4:**
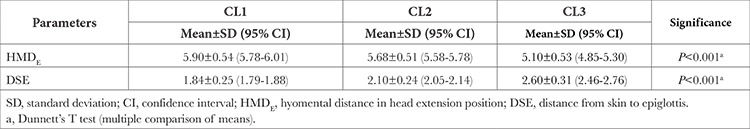
Comparison of HMD_E_ and DSE Parameters in CL 1, 2 and 3 Grade Groups

**Table 5:**
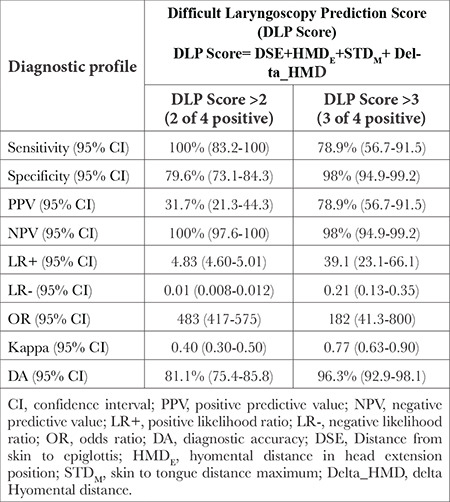
Diagnostic Profile of Difficult Laryngoscopy Prediction Score (DLP Score)
